# Sensitivity Analysis: A Method to Promote Certainty and Transparency in Nursing and Health Research

**DOI:** 10.1177/08445621221107108

**Published:** 2022-06-14

**Authors:** Fabrice I. Mowbray, Donna Manlongat, Meghna Shukla

**Affiliations:** 1 Department of Health Research Methods, Evidence and Impact, 3710 McMaster University, Hamilton, Ontario, Canada; 2 College of Nursing, 2954 Wayne State University, Detroit, Michigan, United States

**Keywords:** sensitivity analysis, quantitative research, nursing research, research methods

## Abstract

Nursing and health researchers may be presented with uncertainty regarding the utilization or legitimacy of methodological or analytic decisions. Sensitivity analyses are purposed to gain insight and certainty about the validity of research findings reported. Reporting guidelines and health research methodologists have emphasized the importance of utilizing and reporting sensitivity analyses in clinical research. However, sensitivity analyses are underreported in nursing and health research. The aim of this methodological overview is to provide an introduction to the purpose, conduct, interpretation, and reporting of sensitivity analyses, using a series of simulated and contemporary case examples.

Nursing and health researchers share a common goal of producing *robust* study findings. Robust findings are insensitive to changes in methodological or analytic assumptions (Thabane et al., 2013), and thus improve confidence in the inferences drawn and context-specific generalizability of study findings (Delaney & Seeger, 2013). Sensitivity analyses are commonly conducted to gain insight and confidence in the decisions, methods, analytics, and measures used (Thabane et al., 2013; Vandenbroucke et al., 2014). Health research methodologists and reporting guidelines have emphasized the value of conducting sensitivity analyses (Hayden et al., 2013; Page et al., 2021; Vandenbroucke et al., 2014), though they are consistently underreported in clinical research (Morris et al., 2014; Vandenbroucke et al., 2014). The aim of this paper is to provide an introductory and pragmatic overview for nursing and health researchers on the purpose, conduct, interpretation, and reporting, of sensitivity analysis using a series of simulated and contemporary case examples.

## What is a sensitivity analysis?

Sensitivity analysis is a method used to evaluate the influence of alternative assumptions or analyses on the pre-specified research questions proposed (Deeks et al., 2021; Schneeweiss, 2006; Thabane et al., 2013). In other words, a sensitivity analysis is purposed to evaluate the validity and certainty of the primary methodological or analytic strategy. Sensitivity analyses are most informative when there is an array of reasonable and differing assumptions (Morris et al., 2014). To illustrate, envision a graduate nursing studentconcerned about the impact of outlier values on the statistical estimates of their regression model. To determine the influence of outlier values, they examine model estimates and associated measures of variance both with (pre-specified) and without outliers present in the analysis. The assumption in this scenario is that the few outlier values in the data have minimal influence on estimates if included in the analysis. If statistical estimates and corresponding measures of variance are similar between models, then confidence can be gained in the robustness of study findings with regard to outlier values (El-Masri et al., 2020). In this scenario, it is recommended that authors report the change in estimates of interest, or lack thereof, with the secondary model included in the document's appendices.

## Timing and benefits of conducting a sensitivity analysis

Sensitivity analyses are conducted *after* the study's primary analyses are completed and conclusions have been made on the results of the primary analysis (Thabane et al., 2013). However, it is best to consider the use of sensitivity analyses during the protocol and study development stage to determine potential uncertainties in the study design after all variables and data analysis approaches have been selected (de Souza et al., 2016; Frey & Patil, 2002). Design of the primary research question should include a clear description of any planned sensitivity analyses, where applicable (e.g., covariate imbalance or protocol deviations) (de Souza et al., 2016). Therefore, the primary analysis should closely follow the pre-determined sensitivity analysis approach to address the impact of the previously identified concerns or uncertainties (de Souza et al., 2016).

There is utility in conducting sensitivity analyses on secondary research questions or post-hoc analytics, though these should be classified and reported as ‘*secondary analyses*’ to avoid confusion with the sensitivity analysis reported for the primary analysis (Morris et al., 2014). This type of sensitivity analysis is often carried out to test the validity of arbitrary or unclear decisions made following protocol publication or data collection. For example, if there is uncertainty regarding the cut-off used to define an exposure or outcome, a sensitivity analysis could be conducted to determine if statistical or clinical significance changes when using a different proposed cut-off for these measures. Recently, a recent systematic review conducted a sensitivity analysis to determine if there was any difference in the choice of cut-off value used in the Clinical Frailty Scale to define frailty, and the influence it had on predicting survival after cardiac arrest (Mowbray et al., 2021).

A lack of evidence-based guidelines or expert consensus regarding a particular methodological decision may necessitate this type of sensitivity analysis (Deeks et al., 2021). If a post-hoc sensitivity analysis results in uncertainty about which statistical model to report, such as in an exploratory study, then it is recommended that the most biologically or theoretically plausible model be emphasized in the reports (Morris et al., 2014).

Data screening, cleaning, and analysis, commonly reveal unanticipated barriers and findings, further highlighting the value of post-hoc sensitivity analyses (Morris et al., 2014). However, these post-hoc analyses require a clear rationale and justification outlined in the *Methods* section of the manuscript, including an explanation of the need for the sensitivity analysis (de Souza et al., 2016). Regardless, whether sensitivity analyses are planned either a priori or post-hoc, both intend to answer: *Are the inferences drawn from the data valid and reliable?* This can be confirmed when consistency in results is noted between the primary analysis and sensitivity analysis (Frey & Patil, 2002; Thabane et al., 2013).

Sensitivity analyses are not only beneficial for researchers and peer reviewers, but they also increase the reader's confidence in study findings by verifying and validating results when unable to meet or demonstrate ideal analytic conditions (de Souza et al., 2016; Frey & Patil, 2002). When a sensitivity analysis suggests that results are not robust or consistent (i.e., results differ greatly from the primary analysis), the researcher must take steps to further investigate the potential source of bias. This is particularly important in clinical research, where findings have the potential to influence health policy development, clinical practice, institutional protocols, and ultimately the care and safety of patients. By thoroughly considering and evaluating study assumptions, one gains confidence in translating and applying study findings to clinical and academic settings.

## Case examples

Sensitivity analyses are conducted across a multitude of scientific disciplines and methodologies. To support knowledge retention and translation, we provide a series of simulated and contemporary case examples to display the utility and versatility of sensitivity analyses in nursing and health research. We provide three examples to showcase how sensitivity analyses can be used across multiple phases of the research process; missing data (data cleaning and screening), clustered data (statistical analysis), and meta-analysis (data synthesis).

### Missing data

A common hurdle in clinical research is the presence of missing data (El-Masri & Fox-Wasylyshyn, 2005). The analytic approach chosen when dealing with missing data must consider both the pattern and influence of missing data points (de Souza et al., 2016; El-Masri & Fox-Wasylyshyn, 2005). Complete-case analysis excludes patients with missing data on one or more variables from statistical estimates (Little et al., 2012; Zhou, 2020). This method is not commonly recommended as its use can reduce statistical power and precision of estimates (Little et al., 2012; Nakai et al., 2014; Zhu, 2014). More importantly, complete-case analysis has the potential to bias estimates if data are missing in a systematic fashion and thus not representative of the entire study cohort (Moons et al., 2015). For example, older adults are commonly excluded from clinical trials given their greater propensity for poor health outcomes (Herrera et al., 2010; Watts, 2012). However, if excluded from analyses, this can distort our understanding of the intervention or association of interest, considering they are the largest user of health services (Gruneir et al., 2018; Rais et al., 2013).

Imputation methods are commonly recommended over complete-case analysis when appropriate (e.g., data missing completely at random), given the reasons previously mentioned. Multiple imputation methods are the preferred imputation technique, over single imputation, as they are more robust, though often more computationally intensive (de Souza et al., 2016; Nakai et al., 2014). Bearing in mind the importance of understanding the context and etiology of missing data (Altman & Bland, 2007), one could conduct a sensitivity analysis to determine the influence of missing data by comparing the statistical estimates and model accuracy in a model with and without imputation (i.e., complete case analysis) (Lee & Simpson, 2014). Study findings are considered robust if sensitivity analyses comparing the complete-case model and multiple imputation model yield similar results (Lee & Simpson, 2014).

### Systematic reviews and meta-analysis

**Figure 1.**
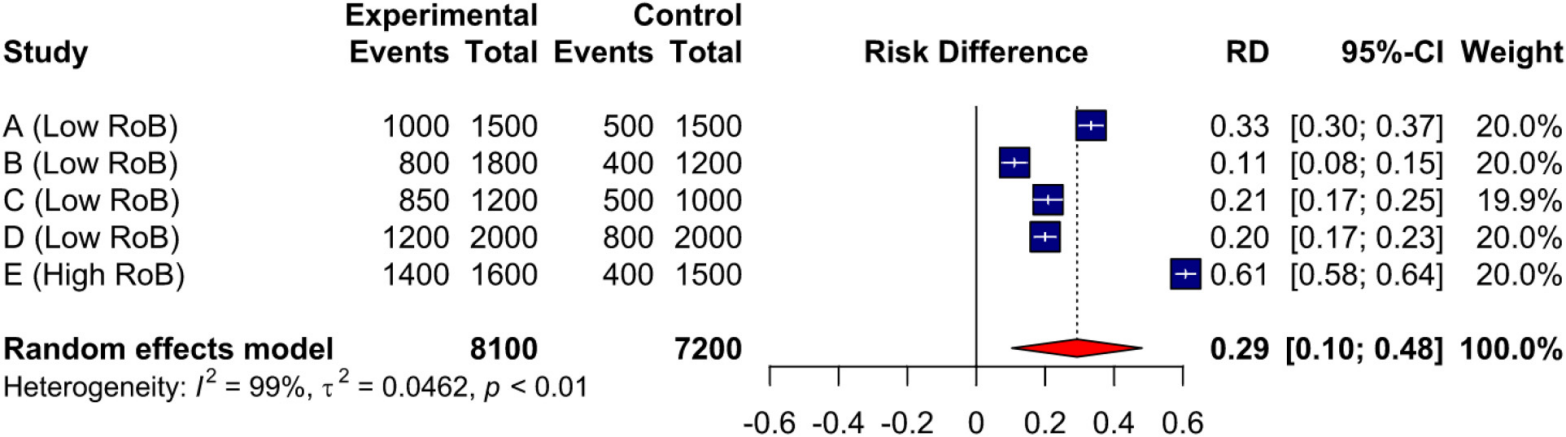
Forest plot with all eligible studies.

**Figure 2.**
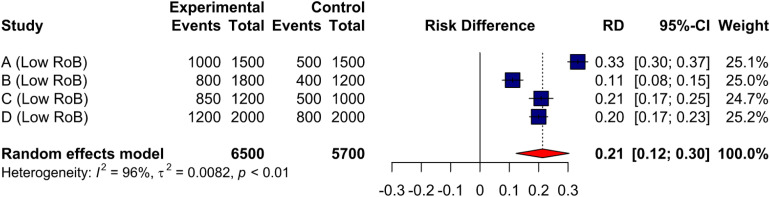
Forest plot with only low risk of bias studies.

Assessing the Risk of Bias (RoB) for studies included in a systematic review is a crucial step to determine the internal validity of individual studies, as well as how threats to validity influence synthesized estimates and reporting (Patole, 2021; Sobieraj & Baker, 2021). To illustrate, imagine one is interested in conducting a systematic review with a meta-analysis aiming to pool data on the effect of enhanced discharge teaching for hospitalized older adults on follow-up with primary care. Figure 1 displays a forest plot with the five eligible studies, four with low RoB, and one with high RoB. To determine if the study with high RoB distorts our pooled estimate of enhanced discharge teaching, one could conduct a sensitivity analysis, in which the high RoB study is removed from the pooled analysis (see Figure 2), and both pooled estimates are compared. As one can see, removing the study with high RoB decreases the absolute risk difference of primary care follow-up by 8% (0.29 → 0.21). This indicates that the high RoB study overestimates the effect of enhanced discharge teaching on primary care follow-up, and thus is likely biasing pooled estimates. While we would typically not pool data with such a high level of heterogeneity (e.g., I^2^) (Foroutan et al., 2020), these figures are purely for educational purposes.

### Correlated (clustered) data

Often in clinical research, we deal with individuals who contribute multiple observations, whereas others may only contribute one (Cleophas et al., 2012; Schober & Vetter, 2018; Senn et al., 2000). Emergency department visitation is an ideal example of this, where some patients may present for multiple repeat visits (Seguin et al., 2018; Slankamenac et al., 2019). If one person (*John*) contributes five visits in a year, and another (*Roopa*) only contributes one visit for the year, analysts would need to take into consideration that *John* contributes approximately five times the information and weight to statistical estimates as *Roopa*, and thus *John* is said to have clustered (or correlated) data. Where clustered observations exist, pooling of these data points may be necessary to facilitate appropriate weighting of estimates and to best capture the variance both within, and between, clusters of data. The five observations contributed by *John* in the prior case example would be an example of one cluster of data (within-cluster variance). The difference in emergency outcomes between *John* and *Roopa* would be an example of between-cluster variance.

Methods exist to handle this clustering in the data, like multi-level modelling and generalized estimating equations (GEE), though the details of these methods are beyond the scope of this paper. With regard to sensitivity analyses, researchers may be interested in testing the influence of using a clustered statistical model versus a standard non-clustered model. If the model associations and measures of variance are similar, then one gains confidence in the decision to report the model without clustering. However, if a clinical or statistical difference is noted, then the authors would be encouraged to reported the clustered model as the primary model. It is recommended that the sensitivity analysis is mentioned in the *Methods* and *Results* sections of the manuscript or report, and any additional analyses are included as supplementary files to support reader decision-making.

## Next steps for nursing and health researchers

The majority of medical journals reflect low usage of sensitivity analyses in most publications of clinical research (Thabane et al., 2013) – the same can be assumed for nursing research. To increase consideration and use of sensitivity analyses in nursing research, nursing and clinical health programs should aim to include education about the utility and importance of sensitivity analyses in graduate level methodology and statistics courses. Students should be encouraged to understand threats to validity (e.g., selection bias, confounding), as this knowledge is necessary to limit and account for possible biases that may invalidate study findings (Altman & Simera, 2010).

Both research and clinical faculty are likely to benefit from understanding the purpose, conduct, and interpretation of sensitivity analyses, as this technique can support the rigour and understanding of their work. Additionally, knowledge of sensitivity analyses can improve RoB assessments during literature appraisals and the peer review process. Encouraging nursing and health researchers to adhere to reporting guidelines (e.g., STROBE, PRISMA) is another strategy to indirectly draw attention to sensitivity analyses, among other analytic strategies (e.g., subgroup analysis) that may further benefit the conduct and reporting of clinical research.

Moving forward, nursing and health researchers should consider the use of sensitivity analyses during the study design phase, and include a priori sensitivity models into research protocols and registries. A discussion of the included sensitivity analyses should also be routinely inserted into funding applications to promote their use with the noble intention of results transparency. The inclusion of sensitivity analyses in these pre-study documents may demonstrate researchers’ thoughtfulness regarding analytic strategy to academic journal editors, funding agencies, and key stake holders.

Reporting the conduct and results of sensitivity analyses, either in-text or in a supplemental file, is recommended to allow readers to easily identify and evaluate any uncertainty in study findings (de Souza et al., 2016). Additionally, highlighting any post-hoc sensitivity analyses, as well as the reasoning for conducting them, is likely to improve contextual understanding of the study for both the researcher and reader. Clinical researchers are encouraged to highlight whether the sensitivity analysis improved certainty of their study findings. They are also encouraged to discuss how the analysis influenced their contextual understanding of the potential biases that influence the research question within the *Discussion* section of respective manuscripts (de Souza et al., 2016).

## Conclusion

Sensitivity analyses can be used in a wide array of analytic scenarios to promote the transparency and validity of proposed research questions and methods. When there is possible uncertainty regarding study methods, analyses, measures, or decisions, conducting and reporting sensitivity analyses is beneficial for both researchers and readership alike. Nursing and other health researchers are encouraged to consider utilizing and reporting the value of sensitivity analyses during the study design phase.
